# High-resolution magnetic resonance elastography reveals differences in subcortical gray matter viscoelasticity between young and healthy older adults

**DOI:** 10.1016/j.neurobiolaging.2018.01.010

**Published:** 2018-05

**Authors:** Lucy V. Hiscox, Curtis L. Johnson, Matthew D.J. McGarry, Michael Perrins, Aimee Littlejohn, Edwin J.R. van Beek, Neil Roberts, John M. Starr

**Affiliations:** a Alzheimer Scotland Dementia Research Centre, University of Edinburgh, Edinburgh, UK; b Edinburgh Imaging facility QMRI, College of Medicine and Veterinary Medicine, University of Edinburgh, Edinburgh, UK; c Department of Biomedical Engineering, University of Delaware, DE, USA; d Thayer School of Engineering, Dartmouth College, Hanover, NH, USA; e Centre for Cognitive Ageing and Cognitive Epidemiology (CCACE), Department of Psychology, University of Edinburgh, Edinburgh, UK

**Keywords:** Brain, Magnetic resonance elastography (MRE), Viscoelasticity, Healthy aging, Subcortical gray matter, Elasticity imaging techniques, Elastography

## Abstract

Volumetric structural magnetic resonance imaging (MRI) is commonly used to determine the extent of neuronal loss in aging, indicated by cerebral atrophy. The brain, however, exhibits other biophysical characteristics such as mechanical properties, which can be quantified with magnetic resonance elastography (MRE). MRE is an emerging noninvasive imaging technique for measuring viscoelastic tissue properties, proven to be sensitive metrics of neural tissue integrity, as described by shear stiffness, μ and damping ratio, ξ parameters. The study objective was to evaluate global and regional MRE parameter differences between young (19–30 years, n = 12) and healthy older adults (66–73 years, n = 12) and to assess whether MRE measures provide additive value over volumetric magnetic resonance imaging measurements. We investigated the viscoelasticity of the global cerebrum and 6 regions of interest (ROIs) including the amygdala, hippocampus, caudate, pallidum, putamen, and thalamus. In older adults, we found a decrease in μ in all ROIs, except for the hippocampus, indicating widespread brain softening; an effect that remained significant after controlling for ROI volume. In contrast, the relative viscous-to-elastic behavior of the brain ξ did not differ between age groups, suggesting a preservation of the organization of the tissue microstructure. These data support the use of MRE as a novel imaging biomarker for characterizing age-related differences to neural tissue not captured by volumetric imaging alone.

## 1 Introduction

The use of medical imaging to identify and quantify brain tissue atrophy (i.e., neuronal cell loss) has been influential in aiding the prediction of onset and progression of many neurodegenerative disorders. Traditional diagnostic magnetic resonance imaging (MRI) is based on the radiologist grading of atrophy, often semiquantitatively, through visual inspection of structural images, whereas research institutes or centers involved in clinical trials, typically use manual, semiautomated or fully automated techniques to study volume changes (i.e., macroscopic size), of regions of interest (ROIs). As an example, the European Medicines Agency has deemed low hippocampal volume an acceptable selection marker for clinical trials of people in the early stages of Alzheimer's disease (AD) (European Medicines Agency, 2011).

Despite the apparent relationship between brain atrophy and clinical syndromes, the association is not simple and linear; atrophy does not necessarily predict clinical symptoms or indeed their severity. Meta-analysis of results from 33 studies found a surprisingly weak positive relationship between hippocampal size and episodic memory ability in older adults, in addition to extreme variability among participants (Van Petten, 2004). One possible reason for this weak relationship is that most age-associated behavioral impairments appear to result from region-specific changes in dendritic morphology, cellular connectivity, axonal integrity, gene expression, or other factors that ultimately alter the network dynamics of neural ensembles that support cognition (Burke and Barnes, 2006, Smith et al., 2004). Accordingly, volumetric MRI is the most basic of neurobiological metrics; a gross proxy of tissue composition and integrity that is not specific to microstructural tissue characteristics. As a result, volumetric measurements are unlikely to characterize presymptomatic neuronal dysfunction, thus limiting the utility of volumetry as a clinical biomarker for the early detection of neurological disorders.

Prior to neurodegeneration, pathological processes, which cause a reduction to, for example, cellular connectivity, are reflected in the biophysical characteristics of brain tissue such as mechanical properties like stiffness and viscous energy dissipation. The mechanical properties of soft tissue may vary over a dynamic range much greater than other physical properties such as magnetic resonance relaxation time (Mariappan et al., 2010), and thus the ability to directly image properties such as tissue stiffness offers the prospect of an imaging technique with high sensitivity. Magnetic resonance elastography (MRE) is being actively developed to noninvasively measure the mechanical properties of the brain in vivo. MRE combines MRI with mechanical wave propagation and records harmonic displacements of soft tissue in MRI phase images using motion-sensitive magnetic field gradients, which are then inverted to estimate underlying viscoelasticity (Muthupillai et al., 1995, Muthupillai and Ehman, 1996). Alterations in the mechanical properties of the brain, therefore, provide a unique contrast mechanism that appears to reflect the integrity of the underlying microstructure and health of brain tissue (Sack et al., 2013). The sensitivity of MRE measures is confirmed by the observation of tissue softening in many neurological diseases (Gerischer et al., 2017, Huston et al., 2015, Lipp et al., 2013, Lipp et al., 2018, Murphy et al., 2011, Murphy et al., 2016, Romano et al., 2014, Streitberger et al., 2012), for a review, see Hiscox et al. (2016) or Murphy and Huston (2017), with animal studies linking this softening to degree of myelin content (Schregel, 2012, Weickenmeier et al., 2016, Weickenmeier et al., 2017), inflammation (Riek et al., 2012), and a reduction in neuronal density related to a decrease in neurogenesis (Freimann et al., 2013, Klein et al., 2014). In general, tissue stiffness parameters likely reflect the composition of the tissue microstructure, whereas viscosity measures, including the phase angle and damping ratio, instead have been suggested to provide information regarding microstructural organization (Sack et al., 2013, Schwarb et al., 2016).

Understanding normal mechanical changes in brain tissue with respect to healthy aging is necessary before determining the efficacy of MRE for neurological disease diagnosis and therapy monitoring. Previous MRE studies into healthy aging have assessed either the global cerebrum (Sack et al., 2009), parcellated slices (Sack et al., 2011), or lobar regional effects (Arani et al., 2015). All studies reveal significant softening to the brain with increasing age, with brain softening occurring at a faster relative rate than brain volume loss with aging (Sack et al., 2011). In contrast, viscosity parameters remain constant suggesting a global preservation of the alignment of the tissue microstructure (Sack et al., 2009, Sack et al., 2011). However, no previous MRE studies into aging have investigated specific neuroanatomical structures, including subcortical gray matter (SGM) ROIs such as the hippocampus. Lying deep within the medial temporal lobes, the hippocampal formation is one of the most studied neuronal systems in the brain due to its implication in memory-specific disorders such as AD and mild cognitive impairment. Rapid improvements of MRE imaging protocols have now transitioned MRE into a high-resolution technique, capable of acquiring whole-brain MRE displacement data at an isotropic resolution of 1.6 mm to enable the study of small brain structures (Johnson et al., 2014). Aging effects have also never been studied with nonlinear inversion (NLI); formulated around a finite-element implementation of the full viscoelastic wave equation, NLI allows for local inhomogeneity and wave reflection effects (McGarry et al., 2012, Testu et al., 2017).

In this current cross-sectional exploratory study, we aim to use these methodological developments to assess the viscoelasticity of the cerebrum globally and 6 SGM matter structures (to include the amygdala [Am], hippocampus [Hp], caudate [Ca], pallidum [Pa], putamen [Pu], and thalamus [Th]), in both young and cognitively healthy older adults. First, we will assess the acceptability of the MRE examination by administering a questionnaire to all participants after the scanning procedure. Second, based on findings from previous work, we predict that the brain will be softer in older adults (i.e., show lower shear stiffness, μ), throughout the cerebrum and all SGM regions. Third, we predict that the global cerebrum will not differ between age groups in its relative viscous-to-elastic behavior (i.e., damping ratio, ξ). It is currently unknown whether age-related differences for ξ will be detected in SGM regions, and thus our analysis is an exploratory one. Finally, we will take into consideration the volume of the cerebrum and each SGM region within our statistical analyses to investigate whether MRE results persist even once ROI volume has been accounted for. MRE results that remain significant after controlling for ROI volume would suggest that MRE parameters provide additive value over volumetric measures alone.

## 2 Material and methods

### 2.1 Participants

Thirty-one apparently healthy participants were recruited from the Join Dementia Research database; 13 were young adult participants aged between 18–30 years and 18 were older participants aged between 65–75 years. Criteria for exclusion included history or current diagnosis of a severe medical, neurological, or psychiatric disorder, history of major head injury, and contraindications for undergoing MRI (such as claustrophobia or the presence of an implanted pacemaker). To ensure older participants, in particular, had no significant underlying memory problems, all were required to complete the Montreal Cognitive Assessment and score within the normal range (>26/30) (Nasreddine et al., 2005). MRE data quality was measured by octahedral shear-strain–based signal-to-noise ratio (OSS-SNR) (McGarry et al., 2011), (see Section 2.4). Overall, 1 young adult was excluded due to OSS-SNR < 3, and 6 older participants were excluded from the analysis: 3 participants had OSS-SNR < 3, 2 participants scored below the required level set for the Montreal Cognitive Assessment, and 1 participant was excluded due to the presence of significant white matter abnormalities, as determined by a consultant radiologist. As a result, the final sample included 24 participants (12 young adults [mean age = 25.2 ± 3.0 years] and 12 older adults [mean age = 69.4 ± 2.5 years]). An equal number of female and male participants were recruited into each group. All participants completed the Edinburgh Handedness Inventory and National Adult Reading Test to measure handedness and IQ, respectively (see Table 1). The study was approved by the National Health Service (NHS) Lothian ethics committee and all study participants gave written, informed consent before the examination.

**Table 1:** Demographic data for participants included in the study

	Young	Older
Number	12	12
Sex	6F/6M	6F/6M
Age (y)	25.2 (19–30)	69.4 (66–73)
EHI	+0.88 (+0.7–1)	+0.86 (+0.3–1)
MoCA	N/A	28.1 (26–30)
NART–full scale IQ	114 (107–121)	123 (115–128)

All values are mean values, with range in parenthesis.

Key: M, male; F, female; EHI, Edinburgh Handedness Inventory; MoCA, Montreal Cognitive Assessment; NART, National Adult Reading Test.

### 2.2 MRI scanning

**Fig. 1:**
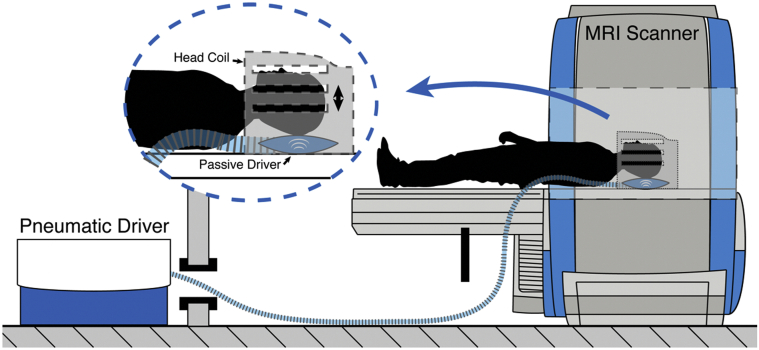
Schematic representation of the experimental pneumatic actuator design for brain tissue vibration (Klatt et al., 2015). Compressed air is transmitted through a plastic tube from an active driver, situated in the MRI control room, to a passive soft pillow-like driver placed beneath the head (Resoundant, Mayo Clinic, Rochester, MN, USA). The vibrations induce a gentle nodding motion of the head.

MRI data were collected using a Siemens 3T Verio whole-body MRI scanner with a 12-channel head receive coil (Siemens Medical Solutions; Erlangen, Germany). The imaging protocol included high-resolution T_1_-weighted and MRE series. T_1_-weighted images were acquired using an MPRAGE sequence (magnetization-prepared rapid gradient echo; 1 × 1 × 1 mm^3^ voxel size; 2400/1000/2.97 ms repetition/inversion/echo times). The MRE acquisition used a 3D multislab, multishot spiral sequence to capture high-resolution displacement data (Johnson et al., 2014). Imaging parameters included the following: 1800/75 ms repetition/echo times; 240 mm square field of view; 150 × 150 imaging matrix; and sixty 1.6-mm thick slices acquired in 10 overlapping slabs. The resulting imaging volume had a 1.6 × 1.6 × 1.6 mm^3^ isotropic voxel size with 96 mm of coverage in the slab direction, which was aligned approximately to the anterior commissure - posterior commissure (AC-PC) line and included the medial temporal lobe. A pneumatic actuator (Resoundant; Rochester, MN, USA) was used to vibrate the brain at a single mechanical frequency of 50 Hz through a soft pad placed below the occipital portion of the head, as shown in Fig. 1. The resulting tissue deformation was encoded using 26 mT/m motion-sensitive gradients embedded in the MRE sequence, which was repeated to capture motion along 3 separate axes with opposite gradient polarities and through 4 phase offsets to observe wave propagation in time. The total MRE acquisition time was approximately 12 minutes.

### 2.3 T_1_ segmentation and mask generation

SGM masks were obtained via automatic segmentation of the T_1_-weighted images using FreeSurfer v. 5.3 through the recon-all pipeline (Fischl et al., 2002). This included skull stripping, automated Talairach transformation, segmentation of the subcortical white/gray matter structures, intensity normalization, automated topology correction, and registration to a spherical atlas. The pipeline generated 6 subcortical masks for the Am, Ca, Hp, Pa, Pu, and Th. All segmentations were visually inspected for accuracy and manual corrections were made when necessary. The T_2_-weighted magnitude MRE images were then coregistered to the structural T_1_-weighted MPRAGE using FMRIB's Linear Image Registration Tool within FMRIB Software Library (Jenkinson et al., 2012). The inverse transform was calculated so that the generated SGM masks from FreeSurfer could be transferred into MRE space to serve as masks for soft prior regularization (SPR), (see Section 2.5), and as ROIs for obtaining quantitative values for each structure. This pipeline is similar to the one used in previous work to separate SGM masks for MRE with SPR; small differences within the pipeline are expected to contribute negligibly to the uncertainty of MRE measurements (Johnson et al., 2016).

### 2.4 Volumetric analysis

The FreeSurfer pipeline generated subcortical volumes in cm^3^ for all 6 ROIs. Estimated total intracranial volume was used to normalize the volume of each ROI for head size using an automated atlas-based head-size normalization pipeline (Buckner et al., 2004). FreeSurfer output, BrainSegNotVent, was used as a measure of total cerebral volume. This ROI includes the sum of the volume of the structures identified in the aseg.mgz volume and the cerebellum, while excluding the brainstem, dura, ventricles (lateral, inferior lateral, 3rd, 4th, 5th), cerebral spinal fluid (CSF), and choroid plexus.

### 2.5 MRE analysis

An OSS-SNR measure was performed to ensure sufficient data quality for stable inversion (McGarry et al., 2011). A data set with an OSS-SNR score < 3 has been excluded from the final analysis due to low displacement SNR (Johnson et al., 2014, Johnson et al., 2016). Non-linear inversion (NLI) (Van Houten et al., 1999, McGarry et al., 2012) was combined with SPR (McGarry et al., 2013) to estimate viscoelasticity from MRE displacement data. The SPR inversion scheme incorporates prior anatomical information to penalize mechanical heterogeneity within an ROI and has previously been shown to improve MRE reproducibility measures (Johnson et al., 2016). Both the MRE displacement data and SGM structure masks in MRE space were input into the NLI algorithm with a weighting of α = 10^−11^, SPR weighting was chosen based on balancing the need to enforce homogeneity, while ensuring convergence. NLI estimates the complex shear modulus, G* = G′ + iG″, from which we determined the shear stiffness, μ and damping ratio, ξ as reported in a number of recent MRE studies (Johnson et al., 2016, Schwarb et al., 2016, Schwarb et al., 2017). Shear stiffness μ, is a composite measure of the shear modulus and determines the wavelength in a viscoelastic solid (Manduca et al., 2001), defined as μ = 2 |G^∗^|^2^/(G′ + |G^∗^|). Shear waves will propagate more quickly through a stiff material (corresponding to a longer wavelength), than through a softer material. Biologically speaking, stiffness values have been associated with neuronal density and neurogenesis (Freimann et al., 2013, Klein et al., 2014), degree of myelination (Schregel, 2012, Weickenmeier et al., 2017, Weickenmeier et al., 2016), inflammation in disease (Riek et al., 2012), as well as functional connectivity (Murphy et al., 2016). Damping ratio, ξ is a dimensionless quantity describing the relative attenuation level in the material, defined as ξ = G″/2G′ (McGarry and Van Houten, 2008), and is similar to the mechanical phase angle often reported in brain MRE (Guo et al., 2013, Lipp et al., 2013). Higher ξ values mean that oscillations created by the shear waves attenuate more rapidly and would suggest that tissue exhibits more viscous fluid-like behavior, as opposed to a more elastic-solid behavior. A less densely connected solid phase, which allows more viscous and frictional losses as tissue constituents slide against each other, is indicative of a reduction in tissue integrity and is expected to relate to the microstructural organization of tissue (Sack et al., 2013).

For illustration purposes only, we coregistered each data set to the Montreal Neurological Institute (MNI152_T1_1 mm) template using Advanced Normalization Tools (ANTS) (Avants et al., 2011) to generate average young and older MRE parameter templates.

### 2.6 Assessment of participant comfort

A questionnaire was administered to participants immediately after the scan, with participants asked to provide a score between 1 and 5 for each of the 3 questions presented. Question (1): Compared to a conventional MRI scan (where there is no vibration), what is your assessment of the discomfort caused by the vibrations required for MRE? Possible answers included the following: 1, severe discomfort; 2, moderate discomfort; 3, mild discomfort; 4, annoyance; and 5, negligible. Question (2): How likely is it that you would take part in the same or a similar MRE study? Question (3): How likely is it that you would recommend this study to other potential participants? For questions (2) and (3), participants were asked to choose from the following options: 1, very unlikely; 2, unlikely; 3, not sure; 4, likely; and 5, very likely.

### 2.7 Statistical analyses

All statistics reported are results obtained in MRE space for each individual. First, a two-way univariate general linear model (analysis of variance [ANOVA]) was used to examine the effect of age group and sex on cerebral (i.e., whole-brain ROI) MRE parameters μ and ξ. Separate ANOVAs were conducted for μ and ξ. Second, a two-way multivariate analysis of variance (MANOVA: Pillai's trace) was used to assess the effect of age group and sex on the combined ROIs including the Am, Ca, Hp, Pa, Pu, and Th. μ and ξ were analyzed separately, and post hoc univariant analyses were summarized. Finally, a univariate ANOVA was used to correct for structure volume, using volume as covariate and age and sex as fixed factors. This was to ensure that changes to MRE parameters were not simply reflecting changes to brain structure volumes. G* power 3.1 (http://www.gpower.hhu.de/en.html) was used to estimate available statistical power π. The calculations show that for a large effect size, of *f* = 0.40, (Cohen, 1969), the power of ANOVA to detect an effect at *p* = 0.05 is π = 0.46. Therefore, we note that the MANOVA is substantially underpowered, resulting in an increased probability of a type II error (false negative). All analyses were performed using SPSS software version 24.0.0 (SPSS Inc., Chicago, IL).

## 3 Results

**Fig. 2:**
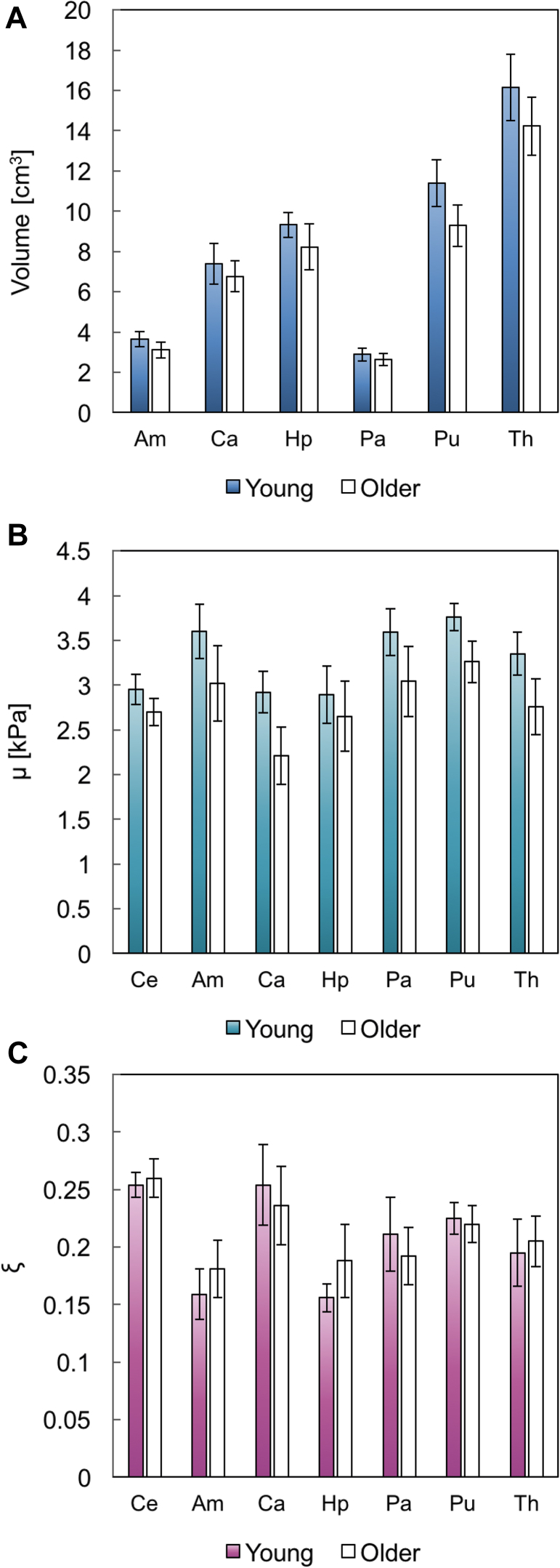
Mean and standard deviation for (A) volume (cm^3^), (B) shear stiffness, μ (kPa), and (C) damping ratio, ξ. N = 12 in each group.

The average OSS-SNR of the brain MRE data was 5.41 ± 1.18 and 6.02 ± 1.67, for the young and older cohorts, respectively, indicating high-quality whole-brain displacement data. Table 2 presents descriptive statistics (mean, standard deviation) for MRI volumetry results and MRE parameters (shear stiffness, μ and damping ratio, ξ), for young and older participants, with data illustrated in Fig. 2. Bilateral values for all 3 parameters were reported as there were no significant left-right hemispheric differences in any ROI for either population (*p* < 0.05).

**Table 2:** Population statistics for MRE parameters and MRI volumetry results

	Ce	Am	Ca	Hp	Pa	Pu	Th
Volume (cm^3^)							
Young	1161 ± 117	3.64 ± 0.37	7.39 ± 1.02	9.32 ± 0.62	2.88 ± 0.33	11.38 ± 1.16	16.15 ± 1.65
Older	1078 ± 89	3.11 ± 0.40	6.77 ± 0.75	8.23 ± 1.13	2.64 ± 0.29	9.28 ± 1.04	14.23 ± 1.44
% Difference	−3.85	−15.70	−8.76	−12.42	−8.70	−20.33	−12.64
μ (kPa)							
Young	2.95 ± 0.17	3.60 ± 0.30	2.92 ± 0.23	2.89 ± 0.32	3.59 ± 0.26	3.76 ± 0.15	3.35 ± 0.24
Older	2.70 ± 0.15	3.02 ± 0.42	2.21 ± 0.32	2.65 ± 0.39	3.04 ± 0.39	3.26 ± 0.23	2.76 ± 0.31
% Difference	−8.47	−16.11	−24.32	−8.30	−15.32	−13.30	−17.61
ξ							
Young	0.254 ± 0.011	0.159 ± 0.022	0.254 ± 0.035	0.156 ± 0.012	0.211 ± 0.032	0.225 ± 0.014	0.195 ± 0.029
Older	0.260 ± 0.017	0.181 ± 0.025	0.236 ± 0.034	0.188 ± 0.032	0.192 ± 0.025	0.220 ± 0.016	0.205 ± 0.022
% Difference	+2.36	+13.84	−7.09	+20.51	−9.00	−2.22	+5.14

Values represent mean +standard deviation.

Key: MRE, magnetic resonance elastography; MRI, magnetic resonance imaging; Am, amygdala; Ca, caudate; Hp, hippocampus; Pa, pallidum; Pu, putamen; Th, thalamus.

### 3.1 MRE acceptability

The MRE component within the scanning protocol was well tolerated, as indicated by the questionnaire scores provided in Table 3. Two participants in the older group felt a moderate level of discomfort (grade 2) from the vibrations generated by the head pillow, whereas 2 participants in both the young and older groups felt a mild level of discomfort (grade 3). An independent samples *t* test found that tolerance of the vibrations was not rated differently between age groups [*t*(22) = −0.713; *p* = 0.484]. In addition, 100% of the younger participants and 92% of the older participants, said it was likely, or very likely, they would take part in a similar study. Finally, 100% of all participants answered that they were likely to recommend that other potential participants take part in the study.

**Table 3:** Subjective assessment of MRE comfort by 24 volunteers

Question	Young	Older
Q1. Vibration (1, severe discomfort; 5 negligible)	4.00 (0.60, 3–5)	3.75 (1.06, 2–5)
Q2. Would return (1, very unlikely; 5 very likely)	4.92 (0.29, 4–5)	4.58 (0.67, 3–5)
Q3. Recommend to others (1, very unlikely; 5 very likely)	4.83 (0.39, 4–5)	4.42 (0.51, 4–5)

Values in parentheses denote the standard deviation and the range.

Key: MRE, magnetic resonance elastography.

### 3.2 Volumetric MRI

#### 3.2.1 Cerebrum

First, a two-way univariate general linear model (ANOVA) was performed to examine the effect of age group and sex on the cerebral volume. There was a statistically significant effect of age on cerebrum volume: [F(1,20) = 5.17, *p* = 0.034], indicating younger participants had larger brain volume than older participants. There was also a significant effect of sex: [F(1,20) = 9.74, *p* = 0.005], indicating larger brain volume in males but no interaction between age and sex (*p* = 0.713).

#### 3.2.2 Subcortical gray matter regions of interest

Second, a two-way MANOVA was performed to assess the effects of age group and sex on volumetric MRI for all 6 SGM structures. There was a statistically significant effect of age [F(6,15) = 4.89, *p* = 0.006] and sex [F(6,15) = 3.10, *p* = 0.035] on the combined ROIs, with no interaction between age and sex, (*p* = 0.966). The univariate effects indicated significant age differences in volume for all individual SGM structures, except for Ca: Am (*p* = 0.002), Hp (*p* = 0.011), Pa (*p* = 0.046), Pu (*p* < 0.001), Th (*p* = 0.004), and Ca (*p* = 0.063). Significant sex differences in volume were found for Ca (*p* = 0.008), Pa (*p* = 0.014), Th (*p* = 0.034), whereas there were no differences in volume for Am (*p* = 0.111), Hp (*p* = 0.518), or Pu (*p* = 0.103).

### 3.3 Shear stiffness μ

#### 3.3.1 Cerebrum

**Fig. 3:**
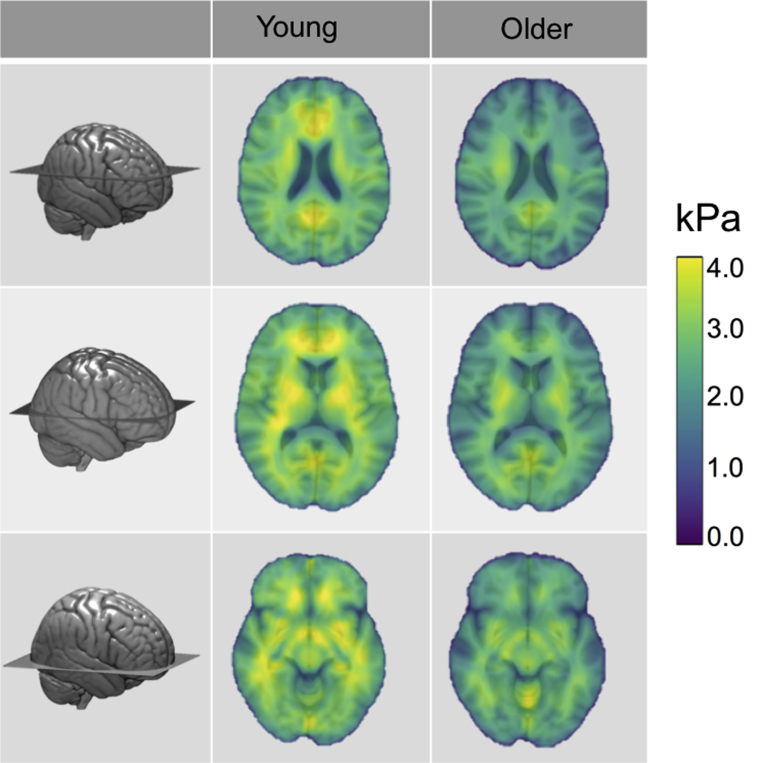
Mean shear stiffness μ properties of the cerebrum (Ce) for young and older adults, showing widespread softer brains in older age (*p* < 0.001). MRE parameter maps have been transformed into standard MNI space, with anatomical information overlaid for illustration purposes. 3D rendering of the MNI template shows the location of the three representative slices. Abbreviation: MNI, Montreal Neurological Institute; MRE, magnetic resonance elastography.

A two-way univariate general linear model (ANOVA) was performed to examine the effect of age group and sex on cerebral stiffness. There was a statistically significant effect of age on Ce μ: [F(1, 20) = 13.33, *p* = 0.002], indicating younger participants had higher cerebral μ than older participants. There was no significant effect of sex (*p* = 0.651) or an interaction between age and sex (*p* = 0.849). Fig. 3 shows 3 representative slices in Montreal Neurological Institute space of Ce μ for both young and older adults.

#### 3.3.2 Subcortical gray matter regions of interest

A two-way MANOVA was performed to assess the effects of age group and sex on μ for all 6 SGM structures. There was a statistically significant effect of age on the combined ROIs, [F(6,15) = 8.75, *p* < 0.001], with no effect of sex (*p* = 0.382), or interaction between age and sex (*p* = 0.408). The univariate effects indicated significant age differences for all individual SGM structures, except for Hp: Am (*p* = 0.001), Ca (*p* < 0.001), Pa (*p* = 0.001), Pu (*p* < 0.001), Th (*p* < 0.001), and Hp (*p* = 0.096).

#### 3.3.3 Volume correction

**Fig. 4:**
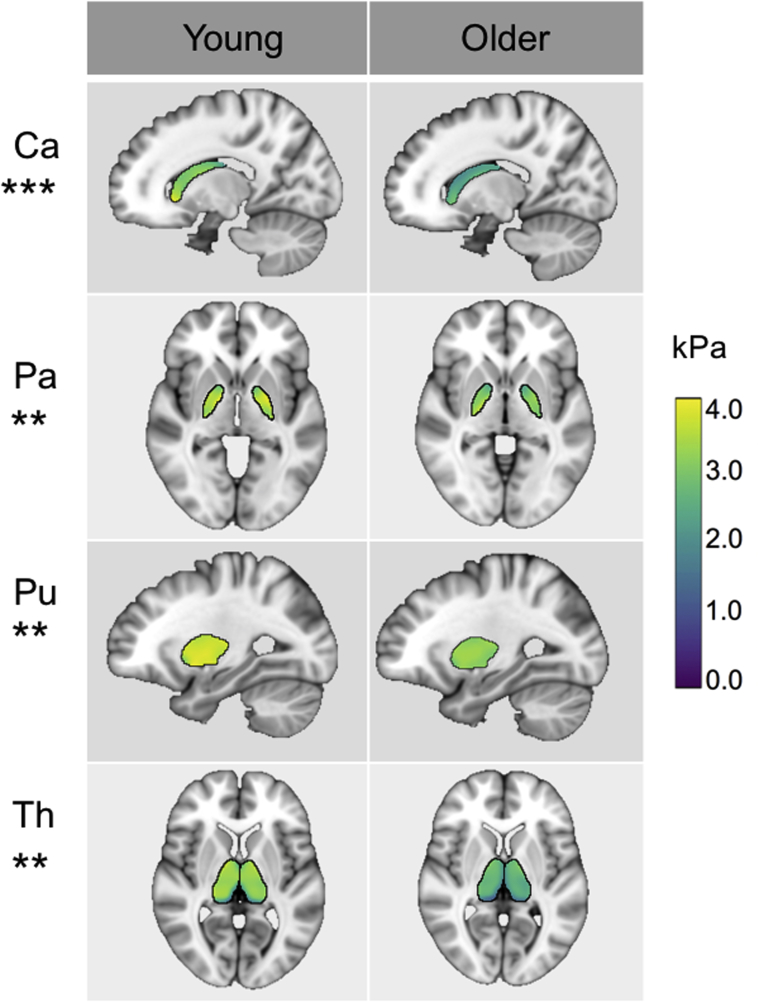
Mean shear stiffness μ properties of SGM structures (Ca, Caudate; Pa, Pallidum; Pu, Putamen; Th, Thalamus) for young and older adults, in standard MNI space. μ of these structures remain significantly different between age groups after correcting for ROI volume, with all being softer in older adults. *** denotes *p* < .001 and ** denotes *p* < .01 significance levels. Abbreviation: SGM, subcortical gray matter; ROI, region of interest.

Third, univariate ANOVA was used to correct μ for volume, using age and sex as fixed factors and volume as a covariate. Each ROI analysis was performed separately. μ of the Ce (*p* < 0.012), Ca (*p* < 0.001), Pa (*p* = 0.002), Pu (*p* = 0.003), and Th (*p* = 0.003) all remained significantly affected by age group after correcting for ROI volume size. Am was no longer significantly different between age groups once Am volume was used as a covariate (*p* = 0.102). Fig. 4 shows mean young and older MRE parameter templates that remained significantly different between age groups after controlling for ROI volume size.

### 3.4 Damping ratio ξ

#### 3.4.1 Cerebrum

Similarly, a univariate general linear model (ANOVA) was conducted to examine the effect of age group and sex on cerebral ξ. There was no significant effect of age (*p* = 0.371), sex (*p* = 0.790), or an interaction between the 2 (*p* = 0.596).

#### 3.4.2 Subcortical gray matter regions of interest

Two-way MANOVA was performed to assess the effect of age group and sex on ξ for all SGM structures. There was a nonsignificant effect of age group on the combined ROIs, [F(6,15) = 1.69, *p* = 0.191], with no significant effect of sex (*p* = 0.827) or interaction between the 2 (*p* = 0.835). No volume correction or post hoc analyses were considered due to the nonsignificant omnibus result.

## 4 Discussion

This proof-of-concept investigation is the first to use our proposed single-frequency, high-resolution, NLI MRE pipeline to assess the effect of healthy aging on the viscoelastic properties of specific neuroanatomical structures in vivo.

First of all, we have demonstrated that the passive MRE driver, consisting of a soft pillow, is well tolerated, even in older adults aged 70 years and over. All participants also completed the study without interruption. To our knowledge, our results provide the first quantitative measure that the MRE head pillow driver is acceptable to participants over a wide age range. This finding is essential when considering the potential clinical utility of MRE as a diagnostic tool. Second, our results indicate that there is significant softening to the Ce, an area largely composed of white matter, and to most SGM structures as a result of increasing age. In older adults, the Ce (8%), Ca (24%), Pa (15%), Th (18%), Am (16%), and Pu (13%) are all significantly softer compared to younger adults. The Hp was the only ROI to not exhibit a statistically significant difference in μ between age groups. Further, μ of all the aforementioned ROIs, excluding the Am, were influenced by age group even after correcting our results to take into account ROI volume. Third, we also demonstrate that there are no differences in Ce damping ratio ξ between age groups, suggesting no overall change in the relative viscous-to-elastic properties of the brain generally. Considering the volumetric MRI analysis independently, the size of the Ce and all SGM structures, except for Ca, is statistically smaller in older adults, as expected.

These results complement previous MRE studies of cerebral aging, which also found that the brain becomes softer with older age (Arani et al., 2015, Sack et al., 2009, Sack et al., 2011). These results are not surprising, given that numerous histopathological studies report that the brain undergoes microstructural and metabolic changes due to normal aging (Callaghan et al., 2014, Eylers et al., 2016, Peters, 2006), and that stiffness is reflective of degree of myelination and neuronal density (Freimann et al., 2013, Klein et al., 2014, Munder et al., 2018). This study, however, is the first to investigate microstructural differences in neuroanatomical regions due to aging. The benefits of this approach enable the investigation of specific brain structures and their associated cognitive functions. Previous work has found a significant correlation between relational memory and the Hp in young adults (Schwarb et al., 2016, Schwarb et al., 2017), whereas more recently, a double dissociation was demonstrated between the orbitofrontal-fluid intelligence relationship and the hippocampal-relational memory relationship (Johnson et al., 2018). These findings provide new opportunities for future investigations to mechanically map the human brain with respect to aging and cognition.

To our surprise, we did not find a reduction in Hp μ in older adults in line with a decrease across other SGM regions. However, the magnitude of the Hp difference (8.3%) is similar to that of Ce (8.5%), which did exhibit a statistically significant difference. Therefore, it is possible that this study was simply underpowered to detect age-related differences to Hp μ: see Section 2.7 for our post hoc power π calculation. Nonetheless, our results are in agreement with a force-indentation study, which compared brain stiffness in healthy young-age to late-middle-age adult mice (human equivalent age of ∼20 years and ∼65 years, respectively), and found no age effect on Hp stiffness (Munder et al., 2018). Our results may reflect the fact that we made reasonable effort to include only cognitively healthy participants; thus our sample may be more resilient to Hp microstructural alterations. This is especially relevant, given a recent study which found that Hp stiffness, determined using multifrequency MRE (MMRE), was lower in patients with AD when compared with healthy older adult controls (Gerischer et al., 2017). Taken together, we can speculate that Hp stiffness may be maintained across the life span until the point of AD neurodegeneration, suggesting that MRE may be a promising noninvasive biomarker for early diagnosis. However, longitudinal investigations will be required to monitor the prodromal stages of disease for the purpose of evaluating whether changes to Hp stiffness can predict clinical decline.

In both age groups, Pu exhibited the highest μ, which complement findings from both Johnson et al. (2016) and Hetzer et al. (2018), who also found the Pu to be the stiffest deep gray matter region, while investigating the Am, Ca, Hp, Pa, Pu, and Th. Hetzer et al. (2018) also further analyzed the perfusion pressure gradient, a measure of cerebral blood flow, and found Pu to display the highest perfusion values, which in turn could predict its stiffness. The distinct mechanovascular properties of Pu, it was argued, could explain the well-known susceptibility of the ROI to hemorrhages. In the younger age group, the Hp was found to exhibit the lowest μ of all of the investigated SGM regions, which agrees with previous MRE findings in young adults (Johnson et al., 2016). In contrast, the Ca was found to be the softest SGM structure in older adults, which is in agreement with Guo et al. (2013), who reported the Ca as being softer than white matter, the Th, and corpus callosum genu, in healthy adults aged between 22 and 72 years.

Considering the damping ratio ξ, we found no difference between age groups for the Ce, which supports our prediction that the brain globally will possess a similarly organized microstructure regardless of age. Our results complement previous work by Sack et al. (2009) and Sack et al. (2011), which found the slope of the complex modulus dispersion (i.e., viscous power-law spring-pot exponent) to remain widely constant throughout the Ce with increasing age, which was attributed to an unaffected geometrical alignment of mechanically relevant structure elements. We also found no significant age-related regional differences in ξ for the 6 selected SGM ROIs. Future region-specific studies with larger sample sizes may find age-related differences in ξ, such as in the Hp. This suggestion is in part driven by the substantial % difference between young and older adults for Hp ξ reported in this study (21%), and the previous finding of a relationship between Hp ξ and relational memory in young adults (Schwarb et al., 2016, Schwarb et al., 2017). In addition, a recent murine study found that the viscous (G″) properties of Hp increase with age (Munder et al., 2018), which the authors suggest may be related to an increase in the number of mobile tissue components caused by age-related brain modifications.

### 4.1 Limitations

The main limitation of this study lies in the small number of participants. This renders our data to limited statistical power and thus an increased probability of a type II error (false negative). Our results, therefore, should be interpreted with caution and replicated in a larger sample. The cross-sectional evidence presented here also cannot test causality between MRE measures and aging, although the current work provides a necessary foundation for future investigations.

MRE inversion is a complex ill-posed problem and as such is under constant development. Due to difficulties in solving the MRE inverse problem, we should acknowledge that other geometrical factors could possibly influence the wave propagation and the quantitative outcomes. Older adults are known to have larger ventricles and a greater degree of CSF due to cerebral atrophy. While we have accounted for ROI volume within our statistical analyses and used conservative masks for our ROIs, we also acknowledge that there may be atrophy within the ROI itself, thus altering the distribution of brain tissue and CSF. As CSF is an incompressible fluid, data model mismatch could cause issues for quantification, although the use of SPR should presumably limit the contribution of CSF-containing voxels. Another concern may be whether our analysis is biased toward the smaller SGM volumes in older adults, as reported in this study. However, previous NLI phantom reconstruction work has been shown to accurately identify changes in stiffness in regions as small as 1 cm^3^ (McGarry et al., 2013). Considering the smallest brain structure measured is the Pa (∼2.5 cm^3^), it is unlikely that volume-related bias is related to our findings of age-related tissue softening.

Another limitation of this study is the inability to directly relate MRE-derived mechanical age differences to an underlying microstructural profile. Most studies that have linked the integrity of tissue microstructure to MRE measurements have used a multiple-frequency acquisition (Freimann et al., 2013, Klein et al., 2014, Riek et al., 2012, Schregel, 2012). MMRE can capture a wide spectrum of experimental results, which are then modeled by a combined viscoelastic element called the springpot model (a combination of the terms ‘spring’ and ‘dashpot’) (Sack et al., 2013). Through these studies, MMRE has been shown to relate the measured dynamics of the complex shear modulus (i.e., powerlaw) to the fractal geometry of structures that build the mechanical scaffold of tissue: that is, changes to spring-pot parameters are associated with the material's complexity, which in turn have been associated with pathophysiological events. While it has been suggested that the powerlaw parameter can be determined from the complex shear modulus at a single frequency (Sinkus et al., 2007), further validation studies are required to investigate whether single-frequency MRE can capture the complex biophysical interactions at the microscopic level. In support of our data, we should mention that complementary age-related findings of brain tissue softening in lobar regions were reported using a single-frequency vibration of 60 Hz (Arani et al., 2015). Nevertheless, the underlying neural substrates causing alterations to mechanical parameters at a single frequency are not fully understood. While tissue stiffness has correlated with neuronal density, demyelination, and levels of inflammation using MMRE, the specificity of this parameter needs further investigation. In addition, the biological correlates for the damping ratio, ξ are less well established, thus ξ currently remains an engineering term in which we can only speculate on its relationship to underlying microstructural alterations.

## 5 Conclusions

In summary, this study is the first to investigate the influence of healthy aging on the viscoelasticity of subcortical neuroanatomical structures in vivo. Novel findings are reported for which older adults displayed globally and regionally specific mechanical brain tissue differences when compared with younger adults. In older age, there is widespread softening (i.e., decrease in shear stiffness μ) of the Ce globally and in all SGM structures (Am, Ca, Pa, Pu, and Th), except for the Hp. However, μ of the Am was no longer influenced by age group once Am volume was used as a covariate. These results suggest that group differences in μ exist even when ROI volume is accounted for and that MRE has additive value over volume. In older age, the brain retains its relative viscous-to-elastic behavior (i.e., damping ratio ξ), both globally and regionally, suggesting a preservation of the organization of the tissue network. These preliminary results suggest that MRE can characterize age-related differences to neural tissue not captured by volumetric imaging alone, and motivate further investigation into the utility of viscoelastic parameters in patients within the clinical or preclinical stages of neurodegenerative disease.

## Disclosure statement

The authors have no actual or potential conflicts of interest.
